# Exploration of Dual Ionic Cross-Linked Alginate Hydrogels Via Cations of Varying Valences towards Wound Healing

**DOI:** 10.3390/polym14235192

**Published:** 2022-11-29

**Authors:** Ernest Man, Dimitrios Lamprou, Claire Easdon, Iain McLellan, Humphrey H. P. Yiu, Clare Hoskins

**Affiliations:** 1 Department of Pure and Applied Chemistry, Technology and Innovation Centre, University of Strathclyde, Glasgow G1 1RD, UK; 2 School of Pharmacy, Queen’s University Belfast, Belfast BT9 7BL, UK; 3 School of Computing, Engineering, Physical Sciences University of the West of Scotland, Paisley PA1 2BE, UK; 4 School of Engineering and Physical Sciences, Institute of Chemical Sciences, Herriot Watt University, Edinburgh EH14 4AS, UK

**Keywords:** polysaccharide, carbohydrate, gallium, calcium, cross-linked, alginate

## Abstract

This study explored the synergistic effects of simultaneously using calcium and gallium cations in the cross-linking of alginate, detailing its effects on the characteristics of alginate compared to its single cation counterparts. The primary goal is to determine if there are any synergistic effects associated with the utilisation of multiple multivalent cations in polymer cross-linking and whether or not it could therefore be used in pharmaceutical applications such as wound healing. Given the fact divalent and trivalent cations have never been utilised together for cross-linking, an explanation for the mode of binding that occurs between the alginate and the cations during the cross-linking process and how it may affect the future applications of the polymer has been investigated. The calcium gallium alginate polymers were able to retain the antibacterial effects of gallium within the confines of the polymer matrix, possessing superior rheological properties, 6 times that of pure calcium and pure gallium, coupled with an improved swelling capacity that is 4 times higher than that of gallium alginate.

## 1. Introduction

Sodium alginate is a well explored substrate that is commonly used as a precursor towards a variety of applications such as wound dressings [1,2], drug delivery devices [3,4,5,6,7,8,9] and in tissue engineering [10,11,12]. In almost all cases, the sodium alginate is processed via cross-linking with multivalent cations (e.g., Ca^2+^ and Mg^2+^) to form a stable insoluble matrix which then allows the solid polymer to be modified towards specific pharmaceutical application.

**Figure 1:**
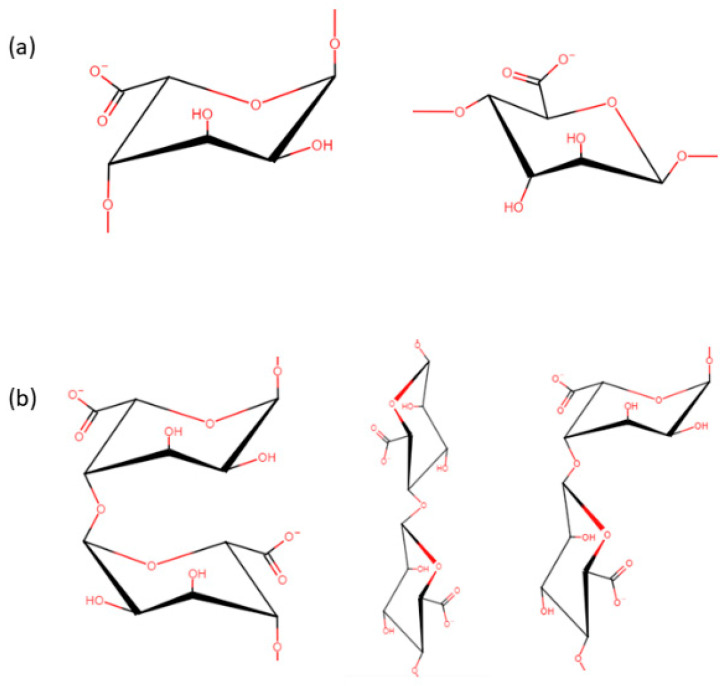
(**a**) α-L-guluronic acid (G) residues and β-D-mannuronic acid (M) residues (**b**) Block configurations. GG MM Mg.

The structural features of alginate can be represented based on the combination of residues that exist, which together as a unit forms a residue block. As shown in Figure 1a,b, the two residue types are α-L-guluronic acid (G) residues and β-D-mannuronic acid (M) residues which are typically found in a homopolymeric series, whereby long chains of GG blocks are observed in tandem with small chains of MM blocks [13]. It should be noted that the variance in G and M residue content within alginate can affect its gelation characteristics, whereby high concentrations of G residue in conjunction with long chains of GG block are responsible for increased rigidity [14], whilst high concentrations of M residues result in more pliable gelation properties. Ultimately when characteristics of both residues are combined to make the MG blocks, the resultant properties then become responsible for flexibility aspect of the polymer matrix [15].

The most common method for cross-linking polymers is through the use of multivalent cations to establish a large network of coordination bonds in conjunction with the lone pairs from the oxygen atoms of the alginic acid chains [16]. The most commonly used cross-linking agents for alginate are calcium cations which can be sourced through ion exchange with compounds that readily dissociate when in solution, i.e., calcium chloride and calcium nitrate. The actual cross-linking process varies in regard to the cation in question, whereby varied cations have different bonding preferences based on the different combination of residue blocks present within the alginate molecule. This was highlighted in the review by Hu et al. [17], which characterises the binding mode and gelation results of alginate through the use of different cross-link ions. The general consensus lies with the fact divalent cations follows the egg-box model that was first termed by Grant et al. [18]. However, different 2+ ions exhibit different forms of preferential selection in terms of its binding priorities, e.g., Ca^2+^ and Sr^2+^ [19,20] binding primarily with G and sometimes MG blocks, Ba^2+^ [21] primarily with G and sometimes M blocks, as well as Cu^2+^ [22] that binds with G and M blocks but displays no preferential bias.

In general, divalent alginate cross-linking is a well explored topic with significant insight in terms of their binding characteristics, but trivalent cations are significantly less explored. Currently the main trivalent ions that have been explored include Al^3+^ [23] and Fe^3+^ [24], where Al^3+^ lacks a detailed mechanism regarding its gelling process, whilst Fe^3+^ has been shown to preferentially form coordination bonds with the carboxyl groups at the end of alginic acid [25]. Overall, the binding energies of trivalent cations have been shown to exceed their divalent counterparts [26]. For example, the weakest trivalent ion La^3+^ displayed a binding energy value of −7.6 to −7.0 MeV, compared to −7.9 to −7.0 MeV for the strongest divalent ion Cu^2+^ [27]. Among the trivalent cation tested in this study (Al^3+^, Sc^3+^, Cr^3+^, Fe^3+^, Ga^3+^, La^3+^), the binding energies have no correlation with their ionic radius. Additionally, there is stronger preferential binding towards to the carboxyl groups instead of OH groups, due to more electrons present in the carboxyl groups and subsequently being transferred to the trivalent cations at a higher priority, relative to the other electronegative groups on the alginic acid structure.

The concept of utilising both divalent and trivalent cations together in one matrix has not yet been looked into and therefore serves as a niche to determine if any synergy exists in such a complex. Given the fact that both divalent and trivalent cations can have preferential binding, this therefore leads to the question of whether or not they can be combined together to fill in the empty residue blocks that are neglected as a result of preferential binding.

In this study, calcium and gallium were selected due to their differing roles in wound healing; combining them into a single cross-linked polymer should theoretically elicit the therapeutic effects of both ions. Calcium partakes in the coagulation cascade leading to effective haemostasis [28]. Regarding wound healing, the contact between the Ca^2+^ loaded polymer and blood/exudate results in an ion exchange event, swapping the calcium ions in the matrix with the monovalent sodium ions in the blood/exudate [29]. On the other hand, gallium (Ga^3+^) has been shown to provide antibacterial activity against common Gram-positive and Gram-negative species such as Pseudomonas aeruginosa, Escherichia coli, and Staphylococcus aureus via the substitution of Fe^3+^ bacterial metabolic pathways [30,31]. Ga^3+^ has also been shown to not elicit any cytotoxic effects against human cutaneous cells [32,33], despite contributing to renal toxicity at high concentrations of around 300 mg/m^3^/d [34]. Ga^3+^ cytotoxicity assays have been tested on human dermal fibroblasts, where the results indicate a slight increase in cell viability for samples containing Ga^3+^ relative to the control and a significantly higher increase in viability after the 3rd day [35].

In the aspect of wound healing, haemostasis, which can be facilitated by Ca^2+^ absorption, is necessary for minimizing total blood loss through the initiation of the coagulation cascade. This also serves to prevent the infiltration of pathogens into the host via the formation of platelet plugs, thereby preventing the accumulation of pathogens which in turn prevents sepsis from occurring. Bactericidal activity also serves an important role in wound healing as pathogens can lead to the increased accumulation of proteases, leading to the cleavage of elastin, which is an integral part of the extracellular matrix [36]. This can then lead to an extended inflammatory phase which in turn retards the transition of the wound into the healing phase, thereby reducing the rate and the quality of regeneration that can occur. All these considerations are necessary for facilitating effective wound regeneration, which is ultimately why Ca^2+^ and Ga^3+^ were chosen as the cross-linking agent.

In this study, we aim to examine if a combined Ca^2+^/Ga^3+^ alginate is mechanistically superior to its mono-cation counterparts. The multi-cation alginate will also be tested to see if it elicits the same levels of antibacterial effect as a pure Ga^3+^ matrix.

## 2. Materials and Methods

### 2.1. Materials

Gallium(III) nitrate hydrate, 99.9% (metals basis) (Alfa Aesar 32116), sodium alginate powder (sourced from Sigma-Aldrich CAS:9005-38-3), calcium chloride pellets (sourced from Sigma-Aldrich Catalog number: c1016).

### 2.2. Methods

#### 2.2.1. Alginate Hydrogel Fabrication

Sodium alginate was dissolved in deionized water and stirred for 45 min at 80 °C to make a 7.5% (*w*/*v*) ratio solution. Portions of alginate solution (3 mL) were aliquoted into 35 mm Petri dishes which were then stored at −21 °C (LEC Medical Freezer LSFSF39UK). The frozen polymer samples were then freeze dried at 0.3 bar and −52 °C for 72 h in a freeze dryer (Christ Alpha 1-2 LD plus). A total of 18 freeze-dried polymer samples were produced, which were then separated into 3 batches of 6 samples that underwent cross-linking with a different reagent.

#### 2.2.2. Cation Alginate Fabrication

Each freeze-dried alginate sample was immersed in separate 10 mL solutions of 0.1 M gallium nitrate, 0.1 M calcium chloride, 1:1, 2:1 and 1:2 ratio solutions of 0.1 M gallium nitrate and 0.1 M calcium chloride solution for 30 min. The cross-linked polymers were then washed 3 times with deionised water to remove any unbound cations. The samples were denoted as GaA, CaA, 1:1 CaGaA, 2:1 CaGaA and 1:2 CaGaA.

#### 2.2.3. Gel Characterisation

##### Scanning Electron Microscopy (SEM)

All alginate samples were air dried at room temperature to remove all moisture from the alginate matrix. These samples were analysed using a Quanta FEG scanning electron microscope (SEM) under high vacuum at 1.4 kV with a spot size of 40 via the secondary electron detector mode to deduce any changes in topography and pore sizes. Average pore sizes were calculated using imageJ, where 30 randomly selected pores from each sample were analysed for their pore diameters where possible.

##### Thermogravimetric Analysis (TGA) and Differential Scanning Calorimetry (DSC)

Simultaneous TGA/DSC analysis on fully desiccated samples was carried out using a Q600 SDT DSC / TGA (TA Instrument). Prior to analysis, samples were dehydrated using a Christ Alpha 1-2 LD plus freeze dryer under 0.3 bar and −52 °C for a period of 48 h to remove excess water. The samples (5–10 mg) were heated with a heating rate of 5 °C/min from 20 °C to 250 °C under flowing nitrogen (75 mL/min), followed by a 30 min isothermal period, then cooled back to 20 °C at 5 °C/min.

##### Rheology

Samples from each alginate variation were submerged in deionized water for 4 h at room temperature and standard pressure, before undergoing an oscillatory amplitude sweep using a HAAKE MARS rheometer by ThermoFisher between the 0.1 to 100 Pa shear stress at room temperature and standard pressure. Gap height was set at 0.2 mm coupled with a frequency of 1 Hz, alongside a total of 40 time points. This allowed for the determination of the storage modulus G′ and loss modulus G″.

##### Fourier Transform Infrared Spectroscopy

Samples were analysed using an FTIR with the iD5 ATR attachment on a Nicolet iS5 (Thermofisher, UK), which has a resolution of 0.4 cm^−1^. Prior to analysis, samples were freeze-dried on a Christ Alpha 1-2 LD plus freeze dryer at −52 °C and 0.3 bars for 48 h to ensure full desiccation. These samples were then ground down with a pestle and mortar. Background corrections were first undertaken before 16 scans were taken for each sample.

#### 2.2.4. Bacterial Strain Preparation

*Escherichia coli* ATCC 25922 (*E. coli*) and methicillin-resistant Staphylococcus aureus (MRSA) ATCC BAA-1766 were obtained from ATCC, whereby all the bacterial strains were cultured in lysogeny broth (LB) (Miller) Merck L3522 at 37 °C for 24 h in a MIR-H263-PE.

##### Zone of Inhibition

*E. coli* and Staphylococcus aureus inoculums were adjusted to a population density of 106 CFU/mL and then distributed onto two separate lysogeny broth agar plates using a cell spreader. A circular punch was taken from each of the cation alginate samples whilst they were in their hydrated state, immediately after the wash steps that followed the cross-linking process. Five sample disks calcium alginate (CaA), gallium alginate (GaA, 1:1 calcium gallium alginate (1:1 CaGaA), 1:2 calcium gallium alginate (1:2 CaGaA) and 2:1 calcium gallium alginate (2:1 CaGaA) of 0.35 cm diameter were cut for analysis. After the culture was spread onto the agar, the alginate samples were then firmly placed onto the agar plate in an equidistant manner, with a total of 5 different alginate samples per plate. These plates were then incubated at 37 °C in a Panasonic MIR-H263-PE incubator for a total of 24 h before a fine mist of 2.5 mg/mL 3-(4,5-dimethylthiazol-2-yl)-2,5-diphenyl-2H-tetrazolium bromide (MTT) was sprayed over the plate to highlight areas bacterial metabolic activity.

#### 2.2.5. Swelling Capacity, Evaporative Water Loss and Matrix Curvature

Three set of CaA, GaA, 1:1 CaGaA, 1:2 CaGaA and 2:1 CaGaA were freeze dried at 0.3 bar and −52 °C for a period of 72 h in a Christ Alpha 1-2 LD plus freeze dryer to remove all moisture without contorting the morphology of the polymer. The samples were immediately weighed to determine the initial fully desiccated weight. These were then immersed in deionised water for a total of 24 h under standard conditions before being reweighed to determine the swelling capacity. Prior to each weighing process, paper towels were utilised to remove any excess water from the polymer matrix. After the 24 h period, the samples were submerged in deionized water for another 48 h at ambient temperature and pressure. After reaching maximum saturation, polymers were air dried at room temperature and standard pressure for 24 h. Samples were weighed at the start of the experiment and every 2 h up to 6 h before a final measurement at 24 h was undertaken to determine the evaporative water loss. The air-dried samples were then processed via imageJ to determine the change in matrix angle from a flat hydrated morphology.

#### 2.2.6. Inductively Coupled Plasma Spectroscopy (ICP)

Fully desiccated CaA, GaA, 1:1 CaGaA, 1:2 CaGaA and 2:1 CaGaA polymer samples were split into one quarter pieces and submerged in separate vials containing 2 mL 1 mol nitric acid for 24 h. After the first hour had elapsed, a glass rod was used to physically break apart the polymer samples into smaller fragments. The samples were then heated on a hotplate until boiling, where it was then left to cool down to room temperature. A 1 mL acid-digested sample was extracted before undergoing a 1:100 dilution with deionized water. The diluted samples were ejected through a 0.45 µm membrane filter which removed all the undissolved alginate. The samples were then analyzed on a Perkin Elmer Avio500.

## 3. Results and Discussion

### 3.1. Characterisation of Alginate Wafers

#### 3.1.1. Morphology Study Using SEM

**Figure 2:**
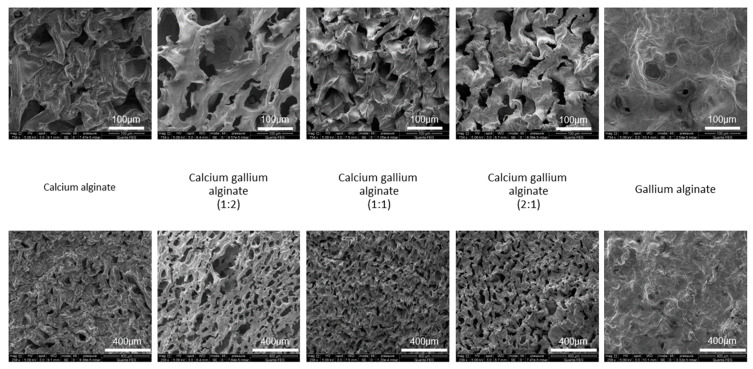
Scanning electron microscopy images of various alginate combinations. Top images: ×734 magnification, lower images: ×238 magnification.

The comparison between each of the five alginate samples is shown in Figure 2 and Table 1, indicating variations in their matrix topography and pore morphologies, which may be indicative of the optimal cross-linker combination.

CaA displayed a wrinkled topography with disordered pattern which is further exacerbated by the highly compact polymer strands, leading to non-uniform pore distribution and smaller pores with respect to the other samples. Regarding the Ca-Ga multi-cation samples, 1:2 CaGaA showed the largest pores with various sizes but they were uniformly distributed. The 1:1 CaGaA sample had a more disorderly topography, where the polymer strands themselves are quite compact with a multitude of folds that have well-defined edges. This sample had the smallest average pore size of 61 μm and the narrowest size distribution among the multi-cation samples. The 2:1 CaGaA sample had the pore size values and standard deviations similar to 1:1 CaGaA; however, these were slightly larger both in terms of their pore size and size distribution. When compared to 1:2 CaGaA, 2:1 CaGaA is still smaller in terms of pore size and standard deviation. GaA has the smallest pore size and the smallest standard deviation amongst all the alginate samples. This sample possessed a flat topography with very few distinguishable features relative to the other cation combinations. The alginate strands here were merged together to create a continuous bulk, with very few identifiable pores. All three combinations of CaGaA possessed uniformly distributed pores with easily discernible topographical features relative to pure CaA and pure GaA.

#### 3.1.2. Rheology Analysis of Samples

**Figure 3:**
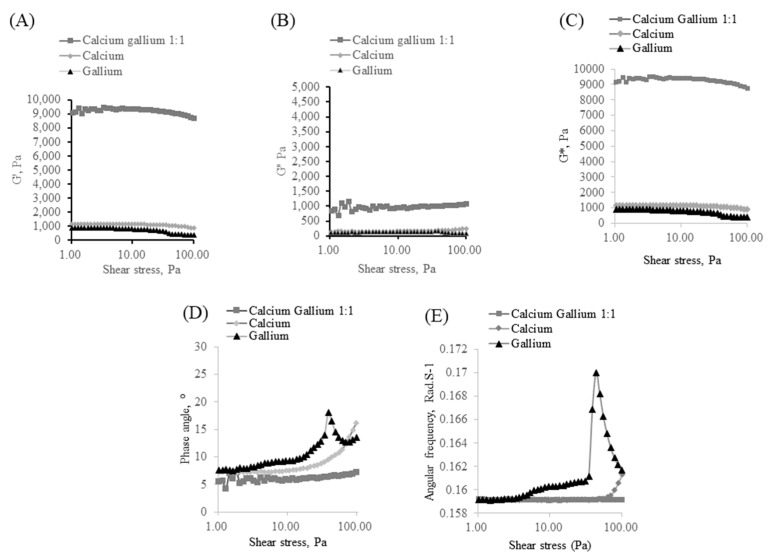
(**A**,**B**) showing the changes in average G’ and G” with respect to increasing shear stress for calcium alginate, gallium alginate and calcium gallium alginate 1:1. (**C**) Comparison of average complex modulus (G*) values between calcium alginate, gallium alginate and calcium gallium alginate 1:1. (**D**) Comparison of average phase angle values between calcium alginate, gallium alginate and calcium gallium alginate 1:1. (**E**) Comparison of average angular frequency values between calcium alginate, gallium alginate and calcium gallium alginate 1:1.

The fabrication of 1:2 CaGaA and 2:1 CaGaA samples produced polymer matrices that exceeded the testing parameters of the equipment and were therefore not suitable for testing. However, the rheology of these samples analysed so as to draw a comparison between all the alginate combinations. As shown in Supplementary Figures S1–S4, 1:2 and 2:1 CaGaA resulted in the occurrence of significant anomalies due to the samples being too rigid, which resulted in both samples exceeding the test parameters of the rheometer. As of such these two samples will not be considered as part of the rheological comparison. Based on the relative comparison of the storage modulus (G′) and the loss modulus (G″) averages, Figure 3A,B, the usage of Ca^2+^ Ga^3+^ solution provides significantly more mechanical stability compared to its single metal counterparts. From this data, it is quite clear, that the combination of Ca^2+^ and Ga^3+^ provides a significantly higher storage modulus than that of using only one metal, thereby implying a higher capacity for deformation energy to be stored elastically. This is further reinforced by Figure 3C, where the comparison of complex modulus (G*) values between the various alginate compositions was shown. The complex modulus quantifies the rigidity of a material below its yield stress and is therefore an important gauge of the material’s mechanical attributes. The obtained complex modulus values indicate that the G* values, as a function of increasing shear stress, for 1:1 CaGaA is approximately 6-fold higher than that of CaA and GaA, thereby suggesting that the combined Ca^2+^/Ga^3+^ may cover the gaps in preferential binding that are associated with single cation population cross-linking.

The comparison of phase angle data in Figure 3D further supports the fact that the combination of Ca^2+^ and Ga^3+^ synergistically boosts the mechanical resistance of the matrix, as the increase in phase angle relative to shear stress is significantly lower than that of GaA or CaA. This is evident by the fact that the maximal increase in phase angle for 1:1 CaGaA alginate is still lower than the vast majority of the values observed in pure gallium and pure calcium. In regard to the average comparison of angular frequency for the three samples, Figure 3E, the maximal change in angular frequency for GaA is 0.010861 Rad.S^−1^ which is the largest, followed by CaA 0.002209 Rad.S^−1^ and 1:1 CaGaA which has the smallest maximal change of 0.000254 Rad.S^−1^. Based on this data, it is quite clear that 1:1 CaGaA has a smaller max change in angular frequency, where the Rad.S^−1^ values of CaA are 8.7 times larger than 1:1 CaGaA and GaA 42.8-fold larger than 1:1 CaGaA. Since angular frequency is a measure of angular displacement per unit of time, it is quite clear that 1:1 CaGaA has the smallest displacement, thereby further supporting the idea of it being the most mechanically stable as a function of increasing shear stress.

The overall implication from this rheological study suggests that there is very strong synergy between Ca^2+^ and Ga^3+^ in terms of their usage together for cross-linking. As explained prior, Ca^2+^ preferentially binds with G blocks and sometimes MG blocks; however, Ga^3+^ does not have any literature that evidences whether or not it has a specific binding preference towards alginate. Based on the rheological data observed from pure Ga^3+^, it may be suggested that the weaker G* values are indicative of preferential binding towards either MM or MG blocks, as Ga^3+^ has higher binding energy than Ca^2+^, which would theoretically lead to significantly higher G* values if it had no preferential binding or if it preferentially bound to GG blocks. This is further supported by the fact that trivalent cations in alginate typically follows a coordination model of an expansive three-dimensional binding network [17], instead of the “egg-box” model, which when coupled with their superior binding energies relative to their divalent counterparts, should result in increased mechanical stability, thus leading to a rheologically stronger matrix. Based on the experimental data, pure Ga^3+^ is approximately the same as, if not slightly weaker, as Ca^2+^, which when considering the binding nature of trivalent cations, should not be the case, unless it preferentially binds to MM or MG blocks. The final piece of evidence supporting the idea that Ga^3+^ is preferential to MM and MG blocks, is the fact that combination Ca^2+^ Ga^3+^ produces a vastly superior G* value which suggests that Ga^3+^ binding preferences do not majorly overlap with the preferences of Ca^2+^. The combination of both Ca^2+^ Ga^3+^ would theoretically occupy the vast majority of bonding points that alginate is comprised of, leading to a much larger G* value with respect to single cation alginate.

**Figure 4:**
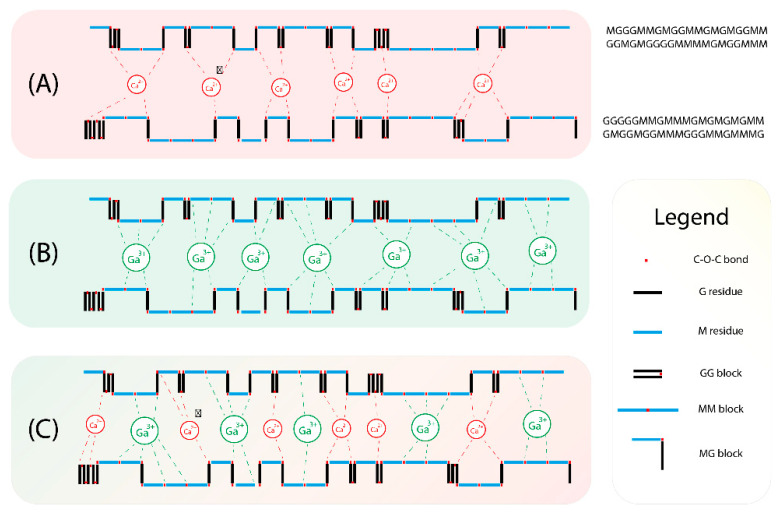
Graphic demonstration of the two-dimensional theoretical binding nature of Ca^2+^ and Ga^3+^ against a randomised alginate chain with a M/G value of 1.09. Both example alginate chains are 20 residues long with the top and bottom chains following the sequence of “MGGGMMGMGGMMGMGMGGMMGGMGMGGGGMMMMGMGGMMM” and “GGGGGMMGMMMGMGMGMGMMGMGGMGGMMMGGGMMGMMMG”, respectively; (**A**) Calcium alginate, (**B**) gallium alginate and (**C**) calcium gallium alginate.

A characterisation study was previously performed on the same batch of sodium alginate to deduce a low M/G value of 1.09, indicating that there are roughly similar quantities of M residues to G residues [37]. With respect to this characterisation result and the binding assumptions of Ga^3+^, it could be implied that the high quantities of G residues are responsible for Ca^2+^ having a higher G’ value than Ga^3+^. This is based off of the idea that the relative abundance of preferential binding points are higher for Ca^2+^, G residues and MG block, whereas Ga^3+^ has a stricter set of preferences, MM and MG blocks. This would then explain why Ga^3+^, despite its higher bonding energies, produced lower G’ values than that of Ca^2+^. The rheological synergy that is observed can also be explained by the relative abundance of G residues. This abundance in G residues would imply an increased likeliness for a single alginate chain to have a large number of Ga^3+^ and Ca^2+^ coordinate bonds at any one time, which when bound to other alginate chains, would then result in a total increase in the number of cation-induced bonds throughout the matrix, Figure 4.

Taking into consideration that 2:1 and 1:2 CaGaA were also used, it could be implied that using a sodium alginate source with a significantly higher or lower M/G value would drastically affect the distribution of Ca^2+^ and Ga^3+^ induced bonds. Given the fact that high M/G values imply a M residue dominant population, such a sample would therefore be more biased towards to Ga^3+^ induced bonding, assuming that the bonding preferences are correct, whereas a low M/G source would therefore expedite Ca^2+^ induced bonding, due to its binding preferences. In this regard, if a high M/G source was utilised, then 2:1 and 1:2 CaGaA would theoretically produce a weaker and stronger matrix, respectively, when compared to an M/G value of approximately 1. The opposite should also hold true, whereby low M/G sources should give stronger and weaker matrices for 2:1 and 1:2 CaGaA, respectively, when compared to an M/G value of approximately 1. Since this concept is purely theoretical, it should be evaluated as a part of the future experiments stemming from this study.

It should be noted that the M/G value of 1.09 would have significantly affected the rheological properties of the matrices, especially with respect to GaA, given the assumptions towards its binding preferences. This was observed during the fabrication step, whereby the GaA samples produced a significantly more brittle matrix compared to its CaA counterpart. Given the M/G evaluation of the alginate source, it is quite clear that the M residue dominant sample would have produced more MM blocks which imparts the pliable characteristics towards alginate. When taking this into consideration, it is quite clear why CaA retains its pliability as none of the MM blocks are bound; however, in the case of GaA, the MM blocks are bound by Ga^3+^ which results in the observable loss in pliability, thus producing a brittle matrix. It was clear that the simultaneous utilisation of two differing cation species in a single matrix produces very significant results. In the specific case of Ga^3+^ and Ca^2+^, the combination was extremely synergistic with respect to the mechanical strength that was imparted to the matrix. One unexpected finding in regard to rheology was the degree of mechanical stiffness that was imparted to the alginate matrix, with respect to CaA and GaA. Since the G* values of 1:1 CaGaA were approximately 6 times that of their singular cation species counterpart, it could be implied that the imparted strength is not additive in terms of the relative contributions by the cations but is instead of a synergistic nature. Theoretically speaking this would not be applicable to the multi cation polymer model if there was competitive binding between the two cation species. This would only occur under the condition that both species had specific binding preferences that overlapped, or if the polymer only had a single point for cation facilitated binding along its macromolecular structure.

#### 3.1.3. Thermal Properties of Alginate Wafers

**Figure 5:**
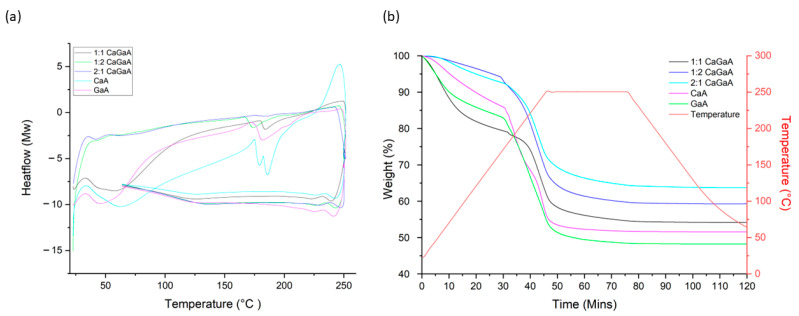
Comparison in (**a**) differential scanning calorimetry (DSC) and (**b**) thermogravimetric (TGA) values for calcium alginate, gallium alginate, calcium gallium alginate 1:1, calcium gallium alginate 1:2 and calcium gallium alginate 2:1.

Initial comparison of all the TGA and DSC data in Figure 5 and Supplementary Table S1, shows significant contrasts in terms of the heat flow and weight loss profiles. For example, there is a variation for the temperature of the endothermic peak observed from the DSC trace, between 166–179 °C, but there are no apparent observable trends. This is likely to be due to the softening or melting of the sample, as well as the possibility of the polymer decomposing. On a more detailed inspection, this endothermic peak in CaA and GaA samples coincide with a significant weight loss event from TGA; this is likely to be due to structural decomposition, such as the dehydration of the -OH groups on the alginate strands. In particular, CaA, this peak splits into a double peak, indicated by two separate events happened close to each other, for example the loss of water from two different -OH groups. It is difficult to pinpoint the identity of these events using DSC/TGA alone. More importantly, this endothermic peak from multi-cation CaGaA samples is smaller in magnitude and it does not directly associate with a significant weight loss event. This is the most obvious for the 1:1 CaGaA sample. Therefore, it could be linked to softening the sample rather than decomposition. Overall, the TGA/DSC analyses for the samples suggested that all samples were thermally stable up to at least 150 °C, with no significant thermal event observed.

For CaA, the endothermic peak occurs at 175 °C with the thermolytic event occurring at 186 °C. GaA on the other hand has its endothermic dip occur at 171 °C and its thermolytic event at 181 °C. When comparing the DSC to the TGA data of GaA, there is a difference of approximately 3.33 °C between the endothermic dip and the sudden weight loss event. The comparison of pure Ca^2+^ against pure Ga^3+^ suggests that Ga^3+^ coordination bonds get thermolytically cleaved at a lower temperature, suggesting weaker overall bond strength, which may support the concept of Ga^3+^ preferentially binding to MM or MG blocks, as these two blocks make up the minority of most residue blocks in alginate.

For the 1:1 CaGaA, the endothermic dip begins 179 °C followed by its thermolytic event at 190 °C, implying high thermal stability. Interestingly when comparing the DSC and TGA data, there is a sharp sudden drop in weight between the endothermic and thermolytic event; however, this stabilises to the same rate of weight loss prior to the endothermic dip. For 1:2 CaGaA, the endothermic dip begins at 166 °C followed by its thermolytic event at 173 °C. The sudden drop in weight loss from the TGA coincides with the start of the endothermic dip, similarly to that of pure CaA. For 2:1 CaGaA, the endothermic dip begins at 176 °C followed by its thermolytic event at 187 °C. The rapid weight loss event from the TGA readings coincide with the beginning of the endothermic dip, similarly to pure CaA and 1:2 CaGaA. It should also be noted that unlike the other samples, this sample had extremely small endothermic dip in regard to its heat flow and thermolytic event.

#### 3.1.4. Fourier Transform Infrared Spectroscopy

**Figure 6:**
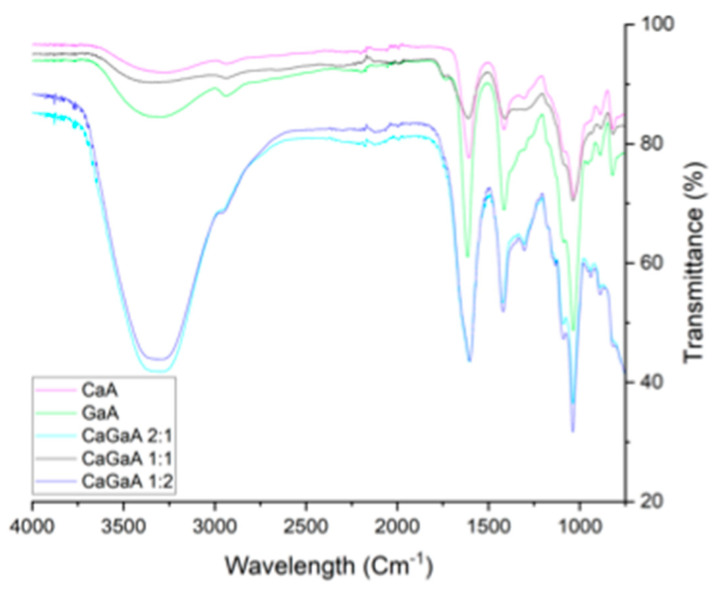
FTIR values between calcium alginate, gallium alginate, calcium gallium alginate 1:1, calcium gallium alginate 1:2 and calcium gallium alginate 2:1.

FTIR analysis of all the samples indicated some significant differences in terms of the relative population of bonds with respect to mono-cation alginate and multi cation alginate, Figure 6 and Supplementary Table S2. The first vibration mode identified is the O-H stretch at 3000–3650 cm^−1^, whereby the OH peaks within the multi-cationic alginates are within the ranges of CaA and GaA. The next stretch is CH anomer occurring between 2850–2980 cm^−1^, whereby the dip is very noticeable for the mono-cation samples but is heavily overshadowed by the OH stretches for 1:2 and 2:1 CaGaA sample. For 1:1 CaGaA, the stretch is not as noticeable compared to mono-cation samples, as the stretch itself is quite shallow which when coupled with an equally shallow OH stretch, reduces its relative visibility. Despite all this, the peak position themselves average around 2937 cm^−1^ and are closely within range of one another without any significant outliers. The COO asymmetric stretch occurs at 1500–1700 cm^−1^ which for all samples are very significant in terms of their identifiability due to the inherent sharpness of this stretch. However, 1:1 CaGaA is an outlier in this regard as it is slightly broader and significantly shorter than the other 4 samples. The relative positions of the peaks are all similar averaging around 1606 cm^−1^, with GaA, 1613 cm^−1^, being minutely shifted upwards with respect to the mean. COO symmetric stretch is the next set of vibration modes which occur at 1350–1500 cm^−1^.

Peaks are mostly sharp and well defined with the exception of 1:1 CaGaA which is very shallow relative to the other samples and lacks a clearly defined range for which the peak begins and ends. The average peak position is 1415 cm^−1^ with no distinct outliers. CCH+OCH occurs at 1250–1350 cm^−1^, which is clearly definable for the samples, 2:1 and 1:2 CaGaA, as well as CaA. In the case of GaA, this specific peak is overshadowed by the COO symmetric stretch but can still be identified to some extent. For 1:1 CaGaA, the peak does not appear at all and is not masked by the COO symmetric stretch, which is in itself very shallow. Generally speaking, all the peaks are in line with one another relative to the average peak position of 1303 cm^−1^ with no distinct outliers. The OCO ring is the next vibration mode occurring at 1050–1100 cm^−1^, presenting itself as a distinctively sharp peak for all 5 samples. The average position of the peak is at 1032 cm^−1^, with no distinct outliers relative to the shifts in wavelength from other functional groups. The final vibrational mode is CO –uronic acid occurring at 920–980 cm^−1^. The peaks themselves are easily identifiable for 2:1 and 1:2 CaGaA, which occur at 940 and 939 cm^−1^, respectively, with 1:1 CaGaA presenting itself as a very small peak relative to the other two multi cationic compositions and is slightly shifted downwards at 934 cm^−1^. For the divalent cation samples, GaA is significantly shifted upwards to give a small peak at 956 cm^−1^, whilst calcium alginate does give any distinct peaks due to it being overshadowed by the OCO ring population. However, taking this into consideration with respect to the observed data, this peak is estimated to occur at 956 cm^−1^.

Overall, the biggest variance amongst this data set, with respect to the chemical shift patterns of all the alginate samples, is 1:1 CaGaA, specifically for the vibrational modes of COO asymmetric, COO symmetric, CCH+OCH and CO –uronic acid. However, in regard to the overall comparison between all samples, the differences in relative peak position were not too substantial. Using CaA as the reference we see that 2:1 CaGaA is shifted upwards for O-H, COO-symmetric and OCO ring by 21 cm^−1^, 7 cm^−1^ and 6 cm^−1^, respectively, whilst CO-uronic acid is shifted downwards by a very 15 cm^−1^. It may be assumed that the presence of Ga^3+^ within a calcium dominant matrix, result in gallium bonding more strongly to the CO groups at the end of the uronic acid residue, thus lengthening the CO bond On the other hand, the comparison of GaA to 1:2 CaGaA indicates a difference in chemical shifts for the groups OH, COO-asymmetric, COO-symmetric and CO –uronic acid. Using GaA as the reference OH, COO-asymmetric and CO –uronic acid are shifted downwards by 4 cm^−1^, 12 cm^−1^ and 17 cm^−1^, respectively, whilst COO-symmetric is shifted upwards by 4 cm^−1^. The assumptions that can be made using GaA as a reference point is the idea that the presence of Ca^2+^ within a gallium dominant matrix may result in the bond lengthening of OH, COO-asymmetric and CO –uronic acid, suggesting that Ca^2+^ binds more preferentially to those specific groups.

#### 3.1.5. Swelling Capacity

**Figure 7:**
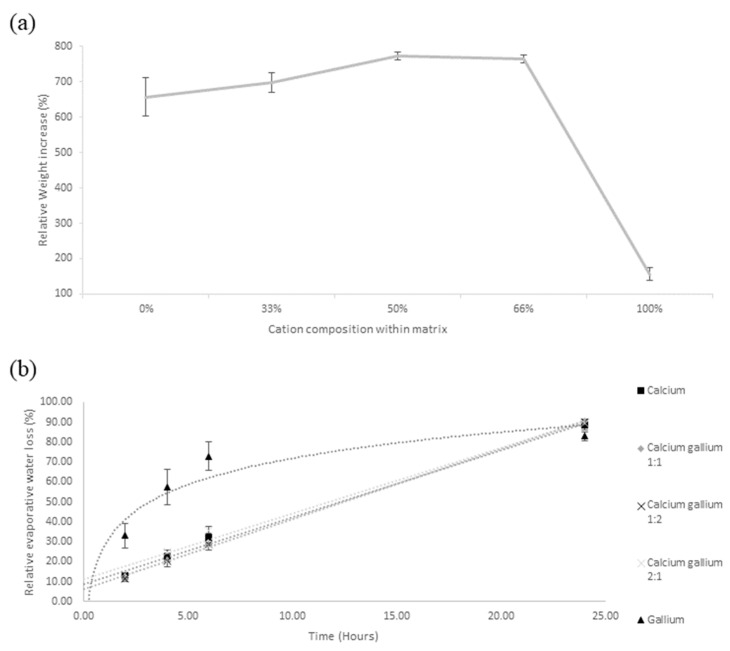
(**a**) Average swelling capacity with respect to the changes in cation composition. (**b**) Average change in evaporative water loss for calcium alginate, gallium alginate, calcium gallium alginate 1:1, calcium gallium alginate 1:2 and calcium gallium alginate 2:1.

Changes in swelling capacity were evaluated, which detail the sample’s capacity to absorb liquid material, respectively. In terms of the changes in swelling capacity, Figure 7a, pure gallium had the lowest swelling capacity, only 155% capacity compared to the other alginate samples with Ca 650–770%, indicating a 4-fold reduction in capacity relative to the other samples. Among these CaGaA samples, the swelling capacity gradually drops from 765% to 657%. The reasoning for such results lies in the smaller average pore size of the sample, Table 1, and may also be affected by the bonding patterns as implied by Figure 4. Given the fact that pure GaA has the lowest swelling capacity values, it could then be implied that the reduced affinity to water may be as a result of the porosity being too low, thereby preventing water from entering the matrix, or it may be that more OH and COOH bonds are being occupied by the coordination bonding of Ga^3+^. As more OH and COOH bonds become occupied by the new coordination bonds, less of them are able to hydrogen bond with water, thus reducing the overall ability of the matrix to hold water.

#### 3.1.6. Evaporative Water Loss and Matrix Curvature

The results from Figure 7b and Table 2 indicate that GaA has the highest initial rate of water loss; however, this slowed down after the 6th hour, thereby implying that GaA’s ability to retain matrix-bound water may be limited by its low porosity, thereby suggesting that the majority of the bound water is retained on the polymer surface.

Given the fact that the other four alginate combinations all possess a linear trendline with a R2 value close 1, with an evaporative water loss rate of 3.29% to 3.53% per hour, it can be ascertained that the presence of Ca^2+^ induced bonds within an alginate matrix can greatly influence the water retention characteristics of the polymer, especially in regard to the trend of evaporative water loss. Given the fact that all other alginate combinations, excluding pure gallium, follow a linear evaporative water loss trend, it can be assumed that the matrices of these samples retained a large enough quantity of water to undergo consistent evaporative water loss during the 24 h period. Taking into account the fact that the average water loss between all samples, excluding GaA, is 0.091 g per hour with respect the atmospheric moisture of the lab, it can be assumed that the large majority of water content within the GaA had been removed within the first several hours. In conjunction with the GaA results from the swelling capacity study, it can be assumed that by the 4th hour, the total amount of water within the matrix was no longer enough to maintain a high-water concentration gradient between the matrix and the lab atmosphere, thus leading to reduced rates of evaporation as the concentrations equalised.

The resulting polymer morphologies from the evaporative water loss experiment also presented a visible trend in regard to the macrostructural matrix curvature, Supplementary Figures S5 and S6. Analysis of the macrostructural curvature between all the alginate combinations, signifies a trend of increasing curvature with respect to increasing Ca^2+^ composition. Based on the results, it is quite clear that the presence of Ga^3+^ within an alginate matrix can partially mitigate the contractual forces elicited by alginate strands as they slowly dehydrate via open air evaporation. These contractual forces are not observed in freeze drying as the water is sublimed away from the frozen state, thereby preventing evaporative forces from acting on the polymer strands. Whilst it is unknown what type of structural resistance each type of cation provides to the structural integrity of the polymer; it can be assumed that it is as a result of the bonding characteristics of specific to each cation. Given the fact that trivalent cations have a 3-dimensional bonding characteristic [17], it could be assumed that Ga^3+^ is able to spatially lock the alginate blocks through mass coordination bonding per cation, thus preventing blocks from moving when other physical forces are being applied. On the contrary Ca^2+^ may only be able to lock the alginate blocks in a 2-dimensional manner relative to Ga^3+^, which would imply that at least 1 dimensional plane would be susceptible to internally and externally applied mechanical forces, thus resulting in the trend displayed in Supplementary Figure S5.

### 3.2. Inductively Coupled Plasma Spectroscopy (ICP)

The ICP data, Table 3, demonstrates the relative presence of cross-linker cations within the alginate matrix of each sample. Based on the observed values of Table 3, the 2:1, 1:1 and 1:2 CaGaA samples indicate a substantially higher concentration of calcium ions relative to that of gallium ions. The relative ion transfer from the cross-link solution into the alginate matrix is represented by Table 3, which signify total percentage transfer of cations from the 10 mL 0.1 mol cross-link solution into the alginate matrix. As shown in Table 3, the transfer of calcium ions is significantly higher than that of gallium for every combination sample. Given the fact that the samples were freeze-dried prior to cross-linking, the cross-linking solutions should therefore permeate evenly and quickly throughout the matrix, which would theoretically result in high levels of ion transfer into the matrix. However, this was mitigated by the pure gallium solution which drastically reduced the pore size, thereby restricting the permeation of Ga^3+^ into the alginate matrix. This restriction resulted in the outer layer of alginate being cross-linked only, which lead to a total absorption of 0.018 g/mL and a relatively low total ion transfer percentage of 7.4%. This cross-link induced pore restriction does not apply to the pure calcium solution which therefore allows the solution to permeate more thoroughly throughout the matrix resulting in a significantly higher ion uptake of 0.088 g/mL which equates to a 79.3% ion transfer percentage.

In regard to the combination samples, it is quite clear that the cation absorption quantities do not reflect the ratios of the external cross-linking solutions 2:1, 1:1 and 1:2 CaGaA. Based on the observed values, 2:1 CaGaA gave a ratio of 66:7 was the highest Ca^2+^:Ga^3+^ ratio; however, it was 1:2 CaGaA which gave the next highest Ca^2+^:Ga^3+^ of 71:18. This results in an approximate ratio of 9 Ca^2+^ ions per 1 Ga^3+^ ion and 8 Ca^2+^ ions per 1 Ga^3+^ ion, respectively. The lowest ratio of Ca^2+^: Ga^3+^ is presented by 1:1 CaGaA, giving a 71:18 ratio, which is approximately 4 Ca^2+^ ions per 1 Ga^3+^ ion. Given the fact that all these combinations displayed a higher Ca^2+^ concentration than Ga^3+^, it may suggest that the presence of Ca^2+^ can limit the total amount of coordination bonds formed by Ga^3+^ and thus reducing the total amount of Ga^3+^ present in the matrix. It may be implied that at the end of the cross-linking period, the majority of the Ca^2+^ were able to form the minimum number of coordination bonds needed to remain stably bound within alginate, whilst some Ga^3+^ were unable to achieve the necessary coordination bonds to remain stably bound to the matrix, leading to its removal during the wash step. Given the fact that Ca^2+^ has less requirements than Ga^3+^ to remain stably bound within alginate, it may then be assumed that the accumulation of bound Ca^2+^ is able to provide enough electrostatic repulsion to prevent Ga^3+^ from fully stabilizing whilst it attempts to form coordination bonds with the alginate.

It should be noted that during the 24 h digestion process only CaA was fully dissolved, whilst the other samples were only partially dissolved. Given the relative bonding strengths of Ca^2+^ compared to Ga^3+^, it may be suggested that the acid digestion process is able to break down the Ca^2+^ coordination bonds more effectively compared to that of Ga^3+^ coordination bonds, possibly leading to a post-digested matrix that is primarily Ga^3+^, which in turn may result in a solution that contains higher quantities of Ca^2+^. Given the fact that the solution is passed through a 0.45 µm filter, the undigested alginate chunks, that are presumed to be Ga^3+^ dominant, are removed from the ICP sampling vessel, thereby subtracting it from the analytical process. In this regard, it may be implied that the ICP data of samples containing gallium may not be fully representative of the sample content.

### 3.3. Zone of Inhibition

**Figure 8:**
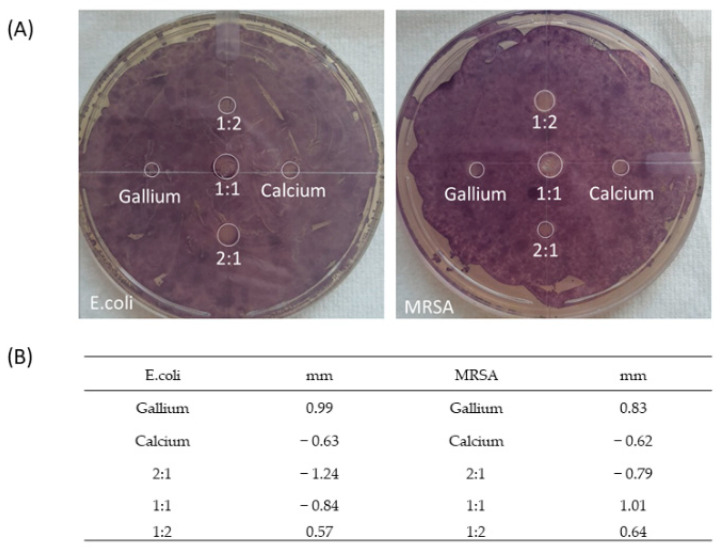
Zone of inhibition combined with MTT to highlight bacterial activity around calcium alginate, gallium alginate, calcium gallium alginate 1:1, calcium gallium alginate 1:2 and calcium gallium alginate 2:1. (**A**) image, (**B**) zone size data.

*E. coli* and MRSA were used to test the antibacterial effectiveness of the cationic alginate samples against Gram-negative and Gram-positive bacteria, respectively, Figure 8. Unlike the standard zone of inhibition which measures the efficacy of an antibacterial agent based on diffusion, which results in the antibacterial zone, the antibacterial effects of the gallium polymers are retained within the matrix and does not steadily diffuse out. As of such, the relative antibacterial efficacy is instead measured by the intrusion of the bacterial colony into the polymer sample, which can be identified through the use of MTT.

Based on the observed data in Figure 8, it is quite clear that gallium by itself elicits antibacterial effect against both Gram-positive and Gram-negative; however, what was not expected, was the fact that the bacterial absorption of Ga^3+^ was substantial enough to disintegrate the entire alginate matrix, leaving an empty zone where the sample used to be. In the case of all other samples, matrix integrity was retained to an extent, thereby limiting the contact between the sample and the inoculum, thus reducing any possible effects that the cations may have on the inoculum. In the case of GaA, the inoculum began to absorb the Ga^3+^ from the matrix, thereby facilitating the breakdown of the polymer matrix, which in turn increased the maximal contact area between the polymer and the inoculum. This gradually resulted in a positive feedback loop, increasing the rate of breakdown for the GaA matrix as more Ga^3+^ was absorbed from it, thereby resulting in increased antibacterial effect at the cost of the matrix’ durability. The breakdown of the matrix confirms the fact that the Ga^3+^ absorption process is facilitated by active transport via the Fe^3+^ substitution route, which is normally utilised in bacterial metabolic pathways [38]. This is supported by the lack of a prominent zone of inhibition outside of the polymer sample’s radius, giving a zone size of 0.99 mm and 0.83 mm against *E. coli* and MRSA, respectively. This suggests that Ga^3+^ does not passively leech into the agar, thus resulting in all the antibacterial activity being retained within the confines of the matrix. It is important that Ga^3+^ does not actively leech out of the matrix, as this may pose the risk of host absorption and therefore systemic circulation which may result in unknown pathological complications. Given the fact that the antibacterial agent, Ga^3+^, is coordination bonded to the alginate molecules, thereby acting as the glue that holds the matrix together, it cannot elicit any antibacterial effect until the matrix has been broken down, where it can then be released and absorbed by the bacteria, thus inducing the mediation of the Fe^3+^ bacterial metabolic pathways.

In regard to antibacterial efficacy of the other cation combinations, 1:1 CaGaA and 1:2 CaGaA appear to be more effective against MRSA when compared to *E. coli*. 1:1 CaGaA and 1:2CaGaA against MRSA possessed a zone size of 1.01 mm and 0.64, respectively, whilst the two combinations against *E. coli* possessed a size of −0.84 mm and 0.57 mm, respectively. Comparisons between the two inoculums suggest that 1:1 CaGaA and 1:2 CaGaA may be less effective against *E. coli* as there in a concentrated ring of purple formazan around the outer perimeter of the 1:1 CaGaA sample, which indicate higher levels of bacterial metabolic activity. It should also be noted that this concentrated ring of purple formazan is also found on calcium and 2:1 CaGaA samples for both MRSA and *E. coli*, giving a zone size of −0.79 mm and −1.24 mm, respectively.

The possible reason for such an occurrence may be due to the presence of Ca^2+^ for both inoculums, where E.coli may have a higher intake of Ca^2+^ relative to MRSA. It has been documented that E.coli requires Ca^2+^ for a certain physiological functions such as the regulation of the bacterial “enzoskeleton” and for its usage to facilitate a “general reset” in bacterial cells [39]. On the contrary there is lack of research regarding the utilisation of Ca^2+^ by MRSA, so it may be possible that MRSA does not require additional Ca^2+^ for major physiological functions and so therefore it does not need to actively absorb it from the polymer matrix. In this regard it may be implied that the higher concentrations of calcium relative to gallium results in 2:1 CaGaA becoming more hospitable for bacterial growth, compared to 1:1 and 1:2 CaGaA which has a higher gallium content, thus resulting in more potent antibacterial activity. This is further reinforced by the fact that pure calcium matrix presented a zone of inhibition size of −0.63 mm and −0.62 mm for *E. coli* and MRSA, respectively, indicating the active intrusion of bacteria into the matrix. Another possible reason may be due to the CaGaA samples having increased structural rigidity, leading to slower breakdown of the polymer matrix, which in turn implies lower rates of Ga^3+^ release and thus a drastic reduction in antibacterial effect. Given the fact that the bacterial colony has been able to intrude some of these samples, it therefore implies that the antibacterial activity is dependent on the breakdown of the polymer matrix, so as to release the antibacterial agent. Taking into account the ICP data, the presence of gallium within the matrix is lower than expected, which will undoubtedly impact the intensity of the antibacterial effect.

Given the fact that the deeper purple of formazan is indicative of increased metabolic activity, it could be surmised that the alginate combinations containing Ca^2+^ had slowly leeched out of the matrix or that the Ca^2+^ was slowly absorbed by the bacterium via active transport. This increase in Ca^2+^ uptake would then lead to the activation of more physiological functions that are specifically associated with Ca^2+^, thus leading to increased metabolic activity. This may be feasible given the fact that both inoculums were grown on lysogeny broth which does not provide Ca^2+^, thereby leading to an increased rate of uptake by the inoculums. As for the reason why this does not apply to MRSA 1:1 CaGaA and 1:2 CaGaA samples, it may be due to the higher concentrations of Ga^3+^ present in the matrix which would be absorbed more readily alongside Ca^2+^ as bacterial metabolic activity begins to increase. This in turn would lead to a build of Ga^3+^ within the bacterium, which would subsequently lead to cell death, thereby ceasing all metabolic activity and preventing an increase in formazan around the matrix. As for the reason why the formazan ring was present around the *E.coli* 1:1 CaGaA samples, it may be due to E.coli being relatively less susceptible to gallium when compared to MRSA, thereby allowing more metabolic activity to occur before eventual cell death.

The implications of these results may be extended towards other combinations of cations, where Ca^2+^ could be replaced with Zn^2+^ to impart further antibacterial activity [40]; however, this may result in faster disintegration of the polymer matrix as the Zn^2+^ will be consumed. In terms of its usage within regenerative medicine, this variability in cation combination may allow for further customisation when designing a polymer matrix. This in turn may allow for further progress towards patient specific treatments as the polymer matrix can be adjusted to suit patient’s needs. Taking into account that Ga^3+^ has a higher binding energy than Ca2^+^ with alginate, it could be theorised that blood/exudate facilitated ion transfer will prioritise the exchange of Ca^2+^ with Na^+^. In a Ca^2+^ only alginate matrix, the replacement of Ca^2+^ with Na^+^ will lead to the eventual breakdown of the matrix, resulting in the total loss of all structural integrity. On the contrary, a multi cationic matrix such as Ca^2+^ Ga^3+^ alginate would not result in the loss of all structural integrity as the total replacement of Ca^2+^ with Na^+^ would still leave the majority of the Ga3^+^ intact, which would theoretically only result in the partial weakening the integrity of the matrix. Hypothetically speaking when this multi cationic alginate is applied onto a wound, the Ca^2+^ would partake in ion exchange resulting in increased haemostasis. An additional benefit would be the fact that the gradual replacement of Ca^2+^ would lead to the partial weakening of the matrix’s structural integrity, thereby letting it conform better to the shape of wound, but also helping it to facilitate the localised release of the Ga^3+^, which will improve its antibacterial efficacy. However, further studies will need to be conducted so as to study the effects of active Ca^2+^ ion exchange within a wound and how it affects the release of Ga^3+^ and its associated antibacterial activity.

## 4. Conclusions

The primary implication of this preliminary study highlights the fact that the simultaneous cross-linking of alginate by cations of different valency, can impart varying degrees of characteristics that are specific to one cation. This sets the framework for increasing the modifiability of polymers during the fabrication stage, where specific physical and non-physical characteristics can be imparted into the matrix with respect to the application at hand.

From the results of this study, it can be concluded that the simultaneous combination of calcium and gallium cross-linkers can produce synergistic effects relative to the characteristics of pure CaA and pure GaA. In terms of its physical characteristics CaGaA displayed superior rheological values, increased water swelling capacity and more consistent porosity relative to single cation alginates. It should also be noted that all three combinations of CaGaA are able to inherit the favourable characteristics of one particular cation. Such examples would include the superior water absorption capacity of CaA against the inferior GaA and the antibacterial properties of GaA compared to the non-antibacterial properties of CaA. Undoubtedly the concept of favourability in this regard is specific to the application at hand; however, by successfully proving the viability of this concept, it therefore implies that other combinations of polymers and cations can also be used to create matrices for even more specific applications, i.e., personalised medicine. Such studies for personalised medicine may explore the synergy between the drug release of therapeutic cations such as Ca^2+^ for blood coagulation [28], Zn^2+^ for the immune system [41], Mg^2+^ for the prevention and treatment of hypertension [42] and Fe^2+^ as supplement against iron deficiency [43].

Overall, it could be surmised that the factors affecting the mechanical structure of a multi-cation polymer matrix include the preferential binding characteristics of the cations, whether their binding preferences overlap and if so, how significant the overlaps are, as well as the binding sites of the polymer with respect to its macromolecular arrangement. These are an important set of points that should be considered given the fact that all polymer applications have some level of dependency on physical characteristics, i.e., polymer mediums for drug delivery, material science, polymer mediums for wound healing. Future experiments should aim to identify whether specific cations and polymers can be utilised together to mitigate mutual or singular characteristics that would be considered a weakness in regards their final application.

## Figures and Tables

**Table 1:** Pore size and alginate strand angle measurements for various alginate combinations.

Alginate	Average Pore Size, μm (±SD)
Calcium	79.1 (28.9)
Calcium gallium 1:2	83.9 (60.5)
Calcium gallium 1:1	61.0 (18.2)
Calcium gallium 2:1	64.7 (21.1)
Gallium	35.6 (14.5)

**Table 2:** Changes in average evaporative water loss.

	Evaporative Water Loss (%)			
Cross-linker combination	2 h	4 h	6 h	24 h
Gallium	33.11	57.50	72.92	82.88
Calcium	12.82	22.49	32.32	88.51
Calcium gallium 1:1	11.26	20.83	28.81	90.52
Calcium gallium 1:2	11.32	20.18	28.88	90.05
Calcium gallium 2:1	13.93	24.76	35.91	89.50

**Table 3:** (A) Cross-link cation quantities and ratios within the alginate matrix. (B) Relative ion transfer from external ion source into the alginate matrix.

**(A)**	**Solution Concentration**	**Calcium (g/mL)**		**Gallium (g/mL)**		**Ratio (Ca:Ga)**
	100% Calcium	0.088		0		N/A
	66% Calcium 33% Gallium	0.053		0.006		66:7 (9.4:1)
	50% Calcium 50% Gallium	0.057		0.014		71:18 (3.9:1)
	33% Calcium 66% Gallium	0.074		0.001		93:12 (7.8/1)
	100% Gallium	0		0.018		N/A
**(B)**	**Solution concentration**		**Calcium (%)**		**Gallium (%)**	
	100% Calcium		79.3		0	
	66% Calcium 33% Gallium		47.6		2.2	
	50% Calcium 50% Gallium		51.2		5.8	
	33% Calcium 66% Gallium		67.		3.8	
	100% Gallium		0		7.4	

## Data Availability

Data is available upon request and held on the University of Strathclyde central repository.

## Supplementary Materials

The following supporting information can be downloaded at: https://www.mdpi.com/article/10.3390/polym14235192/s1, Figure S1: Average phase angle values between calcium alginate, gallium alginate, calcium gallium alginate 1:1, calcium gallium alginate 1:2 and calcium gallium alginate 2:1.; Figure S2: Average complex modulus (G*) values between calcium alginate, gallium alginate, calcium gallium alginate 1:1, calcium gallium alginate 1:2 and calcium gallium alginate 2:1; Figure S3: Average complex modulus (G*) values between calcium alginate, gallium alginate, calcium gallium alginate 1:1 and calcium gallium alginate 2:1; Figure S4: Average angular frequency values between calcium alginate, gallium alginate, calcium gallium alginate 1:1, calcium gallium alginate 1:2 and calcium gallium alginate 2:1; Figure S5: Changes in macrostructural curvature as a result of open-air desiccation; Figure S6: Effects of Ca2+ concentration on matrix curvature; Table S1: Endothermic and exothermic events for calcium alginate, gallium alginate, calcium gallium alginate 1:1, calcium gallium alginate 1:2 and calcium gallium alginate 2:1; Table S2: Peak position of FTIR vibrations between calcium alginate, gallium alginate, calcium gallium alginate 1:1, calcium gallium alginate 1:2 and calcium gallium alginate 2:1.

## References

[1] Moura L.I., Dias A.M., Carvalho E., de Sousa H.C. (2013). Recent advances on the development of wound dressings for diabetic foot ulcer treatment-a review. Acta Biomater..

[2] Parani M., Lokhande G., Singh A., Gaharwar A.K. (2016). Engineered nanomaterials for infection control and healing acute and chronic wounds. ACS Appl. Mater Interfaces.

[3] Wawrzyńska E., Kubies. D. (2018). Alginate matrices for protein delivery-a short review. Physiol. Res..

[4] Seedat N., Kalhapure R.S., Mocktar C., Vepuri S., Jadhav M., Soliman M., Govender T. (2016). Co-encapsulation of multi-lipids and polymers enhances the performance of vancomycin in lipid-polymer hybrid nanoparticles: In vitro and in silico studies. Mater. Sci. Eng. C.

[5] Moebus K., Siepmann J., Bodmeier R. (2012). Novel preparation techniques for alginate-poloxamer microparticles controlling protein release on mucosal surfaces. Eur. J. Pharm. Sci.

[6] Lipour S., Montaseri H., Tafaghodi M. (2010). Preparation and characterization of biodegradable paclitaxel loaded alginate microparticles for pulmonary delivery. Colloid Surface B Biointerfaces.

[7] Hill M., Twigg M., Sheridan E.A., Hardy J.G., Elborn J.S., Taggart C.C., Scott C.J., Migaud M.E. (2019). Alginate/Chitosan Particle-Based Drug Delivery Systems for Pulmonary Applications. Pharmaceutics.

[8] Ahmed A., Getti G., Boateng J. (2018). Ciprofloxacin-loaded calcium alginate wafers prepared by freeze-drying technique for potential healing of chronic diabetic foot ulcers. Drug Deliver. Trans. Res..

[9] Sarheed O., Rasool B.K.A., Abu-Gharbieh E., Aziz U.S.S. (2014). An investigation and characterization on alginate hydogel dressing loaded with metronidazole prepared by combined inotropic gelation and freeze-thawing cycles for controlled release. AAPS PharmSciTech.

[10] Lee B.H., Li B., Guelcher S.A. (2012). Gel microstructure regulates proliferation and differentiation of MC3T3-E1 cells encapsulated in alginate beads. Acta Biomater.

[11] Mhanna R., Kashyap A., Palazzolo G., Vallmajo-Martin Q., Becher J., Moller S., Schnabelrauch M. (2014). Chondrocyte culture in three dimensional alginate sulfate hydrogels promotes proliferation while maintaining expression of chondrogenic markers. Tissue Eng. Part A.

[12] Jeon H., Kang K., Park S.A., Kim W.D., Paik S.S., Lee S.H., Jeong J., Choi D. (2017). Generation of multilayered 3D structures of HepG2 cells using a bio-printing technique. Gut Liver.

[13] Cao L., Lu W., Mata A., Nishinari K., Fang Y. (2020). Egg-box model-based gelation of alginate and pectin: A review. Carbohydr. Polym..

[14] Kuo C.K., Ma P.X. (2001). Ionically crosslinked alginate hydrogels as scaffolds for tissue engineering: Part 1. Structure, gelation rate and mechanical properties. Biomaterials.

[15] Szekalska M., Puciłowska A., Szymańska E., Ciosek P., Winnicka K. (2016). Alginate: Current use and future perspectives in pharmaceutical and biomedical applications. Int. J. Polym. Sci..

[16] Brus J., Urbanova M., Czernek J., Pavelkova M., Kubova K., Vyslouzil J., Abbrent S., Konefal R., Horsky J., Vetchy D. (2017). Structure and Dynamics of Alginate Gels Cross-Linked by Polyvalent Ions Probed via Solid State NMR Spectroscopy. Biomacromolecules.

[17] Hu C., Lu W., Mata A., Nishinari K., Fang Y. (2021). Ions-induced gelation of alginate: Mechanisms and applications. Int. J. Biol. Macromol..

[18] Grant G.T., Morris E.R., Rees D.A., Smith P.J.C., Thom D. (1973). Biological interactions between polysaccharides and divalent cations: The egg-box model. FEBS Lett..

[19] Wang H., Wan Y., Wang W., Li W., Zhu J. (2018). Effect of calcium ions on the III steps of self-assembly of SA investigated with atomic force microscopy. Int. J. Food Prop..

[20] Zhang X., Wang L., Weng L., Deng B. (2019). Strontium ion substituted alginate-based hydrogel fibers and its coordination binding model. J. Appl. Polym. Sci..

[21] Hassan R.M. (2020). Prospective and comparative Novel technique for evaluation the affinity of alginate for binding the alkaline-earth metal ions during formation the coordination biopolymer hydrogel complexes. Int. J. Biol. Macromol..

[22] Caccavo D., Ström A., Larsson A., Lamberti G. (2016). Modeling capillary formation in calcium and copper alginate gels. Mater. Sci. Eng. C.

[23] Alkhayer G., Khudr H., Koudsi Y. (2020). Spectroscopic and chromatographic investigation of chiral interactions between tiaprofenic acid and alginate–metal-complexes. RSC Adv..

[24] Liu Y., Chen J., Liu Z., Xu H., Shi Z., Yang Q., Hu G.H., Xiong C. (2020). Necklace-like ferroferric oxide (Fe_3_O_4_) nanoparticle/carbon nanofibril aerogels with enhanced lithium storage by carbonization of ferric alginate. J. Colloid. Interface Sci..

[25] Chen Y., Teng J., Liao B.Q., Li R., Lin H. (2020). Molecular insights into the impacts of iron(III) ions on membrane fouling by alginate. Chemosphere.

[26] Menakbi C., Quignard F., Mineva T. (2016). Complexation of Trivalent Metal Cations to Mannuronate Type Alginate Models from a Density Functional Study. J. Phys. Chem. B.

[27] Agulhon P., Markova V., Robitzer M., Quignard F., Mineva T. (2012). Structure of Alginate Gels: Interaction of Diuronate Units with Divalent Cations from Density Functional Calculations. Biomacromolecules.

[28] Singh S., Dodt J., Volkers P., Hethershaw E., Philippou H., Ivaskevicius V., Imhof D., Oldenburg J., Biswas A. (2019). Structure functional insights into calcium binding during the activation of coagulation factor XIII A. Sci. Rep..

[29] Thomas S. (2000). Alginate dressings in surgery and wound management—Part 1. J. Wound Care.

[30] Best M.G., Cunha-Reis C., Ganin A.Y., Sousa A., Johnston J., Oliveira A.L., Smith D.G.E., Yiu H.H.P., Cooper I.R. (2020). Antimicrobial Properties of Gallium(III)- and Iron(III)-Loaded Polysaccharides Affecting the Growth of Escherichia coli, Staphylococcus aureus, and Pseudomonas aeruginosa In Vitro. ACS Appl. Bio Mater..

[31] Hijazi S., Visaggio D., Pirolo M., Frangipani E., Bernstein L., Visca P. (2018). Antimicrobial Activity of Gallium Compounds on ESKAPE Pathogens. Front. Cell. Infect. Microbiol..

[32] Stuart B.W., Stan G.E., Popa A.C., Carrington M.J., Zgura I., Necsulescu M., Granta D.M. (2022). New solutions for combatting implant bacterial infection based on silver nano-dispersed and gallium incorporated phosphate bioactive glass sputtered films: A preliminary study. Bioact. Mater..

[33] Zheng H., Huang Z., Chen T., Sun Y., Chen S., Bu G., Guan H. (2022). Gallium ions incorporated silk fibroin hydrogel with antibacterial efficacy for promoting healing of Pseudomonas aeruginosa-infected wound. Front. Chem..

[34] Chitambar C.J. (2010). Medical applications and toxicities of gallium compounds. Int. J. Environ. Res. Public Health.

[35] Pourshahrestani S., Zeimaran E., Kadri N.A., Gargiulo N., Samuel S., Naveen S.V., Kamarul T., Towler M.R. (2016). Gallium-containing mesoporous bioactive glass with potent hemostatic activity and antibacterial efficacy. J. Mater. Chem. B.

[36] Ohbayashi T., Irie A., Murakami Y., Nowak A.M., Potempa J., Nishimura Y., Shinohara M., Imamura T. (2010). Degradation of fibrinogen and collagen by staphopains, cysteine proteases released from Staphylococcus aureus. Microbiology.

[37] Man E., Oluwasanmi A., Lamprou D.A., Goudie K.J., Liggat J., Hoskins C. (2022). Effect of preparation method on alginate wafer properties. J. Appl. Polym. Sci..

[38] Miethke M., Marahiel M.A. (2007). Siderophore-based iron acquisition and pathogen control. Microbiol. Mol. Biol. Rev..

[39] Onoda T., Enokizono J., Kaya H., Oshima A., Freestone P., Norris V. (2000). Effects of Calcium and Calcium Chelators on Growth and Morphology of Escherichia coli L-Form NC-7. J. Bacteriol..

[40] Almoudi M.M., Hussein A.S., Hassan M.I.A., Zain N.M. (2018). A systematic review on antibacterial activity of zinc against Streptococcus mutans. Saudi Dent. J..

[41] Prasad A.S. (2008). Zinc in Human Health: Effect of Zinc on Immune Cells. Mol. Med..

[42] Romani A.M.P. (2018). Beneficial Role of Mg^2+^ in Prevention and Treatment of Hypertension. Int. J. Hypertens..

[43] Aycicek A., Koc A., Oymak Y., Selek S., Kaya C., Guzel B. (2014). Ferrous sulfate (Fe^2+^) had a faster effect than did ferric polymaltose (Fe^3+^) on increased oxidant status in children with iron-deficiency anemia. Randomized Controlled Trial. J. Pediatr. Hematol. Oncol..

