# Supplementary material for: Exploration of Dual Ionic Cross-Linked Alginate Hydrogels Via Cations of Varying Valences towards Wound Healing
Source: Polymers (Basel). 2022 Nov 29;14(23):5192. doi: 10.3390/polym14235192 (PMC9738749; doi:10.3390/polym14235192)
Supplement: Supplementary file 1 [file polymers-14-05192-s001.zip › polymers-1990608-supplementary.pdf]

Supplementary information

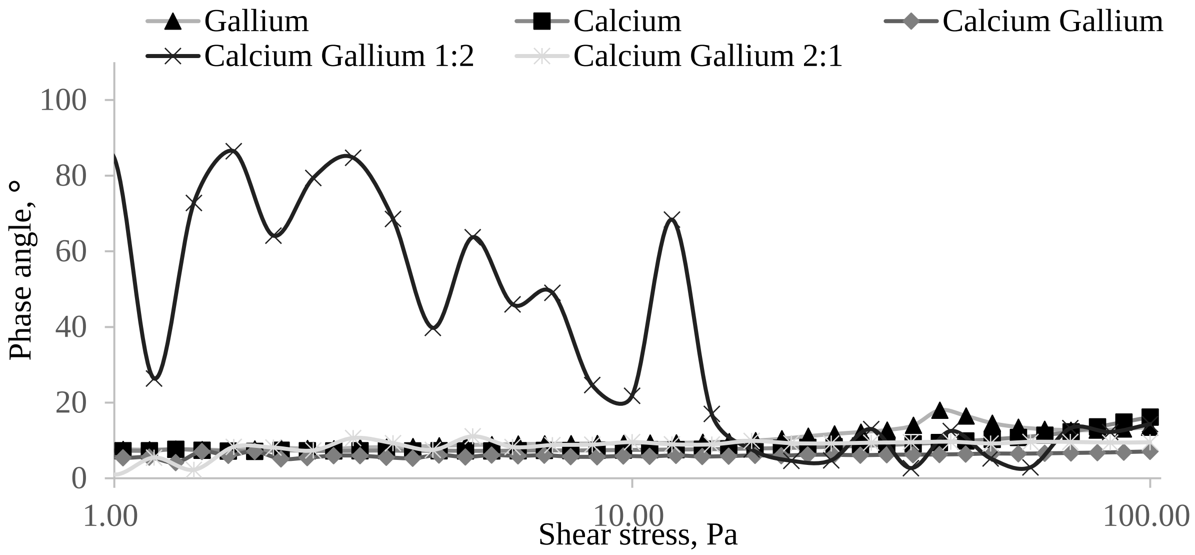

Supplementary Figure S1. Average phase angle values between calcium alginate, gallium alginate, calcium gallium alginate 1:1, calcium gallium alginate 1:2 and calcium gallium alginate 2:1.

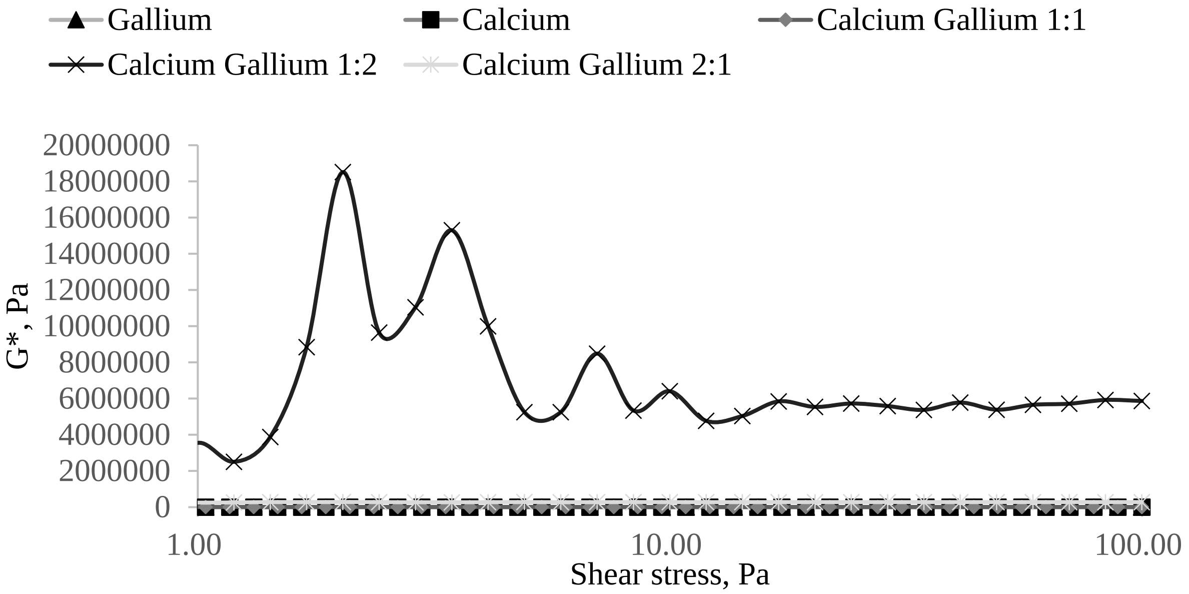

Supplementary Figure S2. Average complex modulus (G*) values between calcium alginate, gallium alginate, calcium gallium alginate 1:1, calcium gallium alginate 1:2 and calcium gallium alginate 2:1.

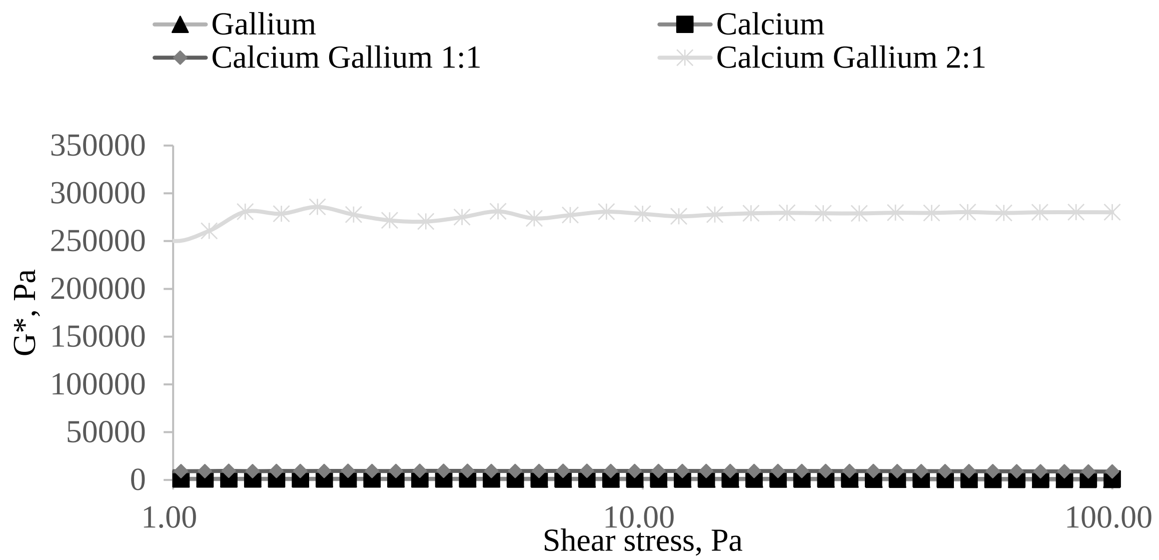

Supplementary Figure S3. Average complex modulus (G*) values between calcium alginate, gallium alginate, calcium gallium alginate 1:1 and calcium gallium alginate 2:1.

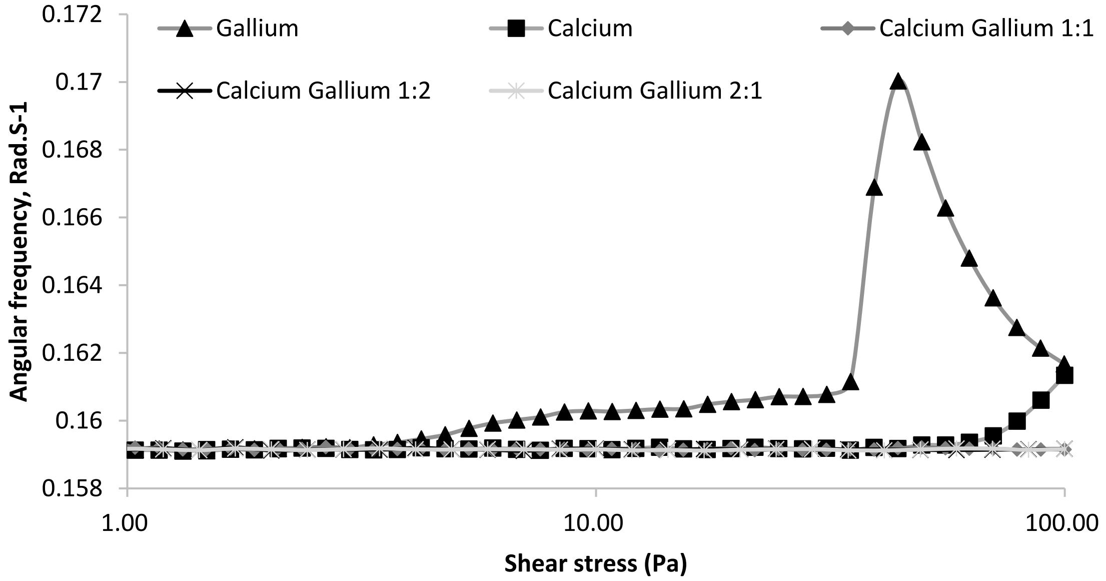

Supplementary Figure S4. Average angular frequency values between calcium alginate, gallium alginate, calcium gallium alginate 1:1, calcium gallium alginate 1:2 and calcium gallium alginate 2:1.

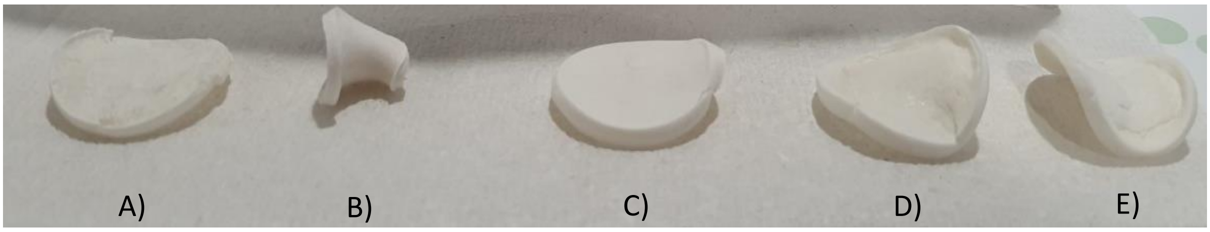

Label	Composition	Ca^{2+} composition %	Average curvature °	Change in curvature ° (±SD)
A	Gallium	0	155.4	24.60 (3.769173)
B	Calcium	100	33.569	146.43 (9.95437)
C	Calcium gallium 1:1	50	127.083	52.92 (3.378464)
D	Calcium gallium 1:2	33.3	139.942	40.06 (4.814503)
E	Calcium gallium 2:1	66.6	77.214	102.786 (9.034421)

Supplementary Figure S5. Changes in macrostructural curvature as a result of open-air desiccation.

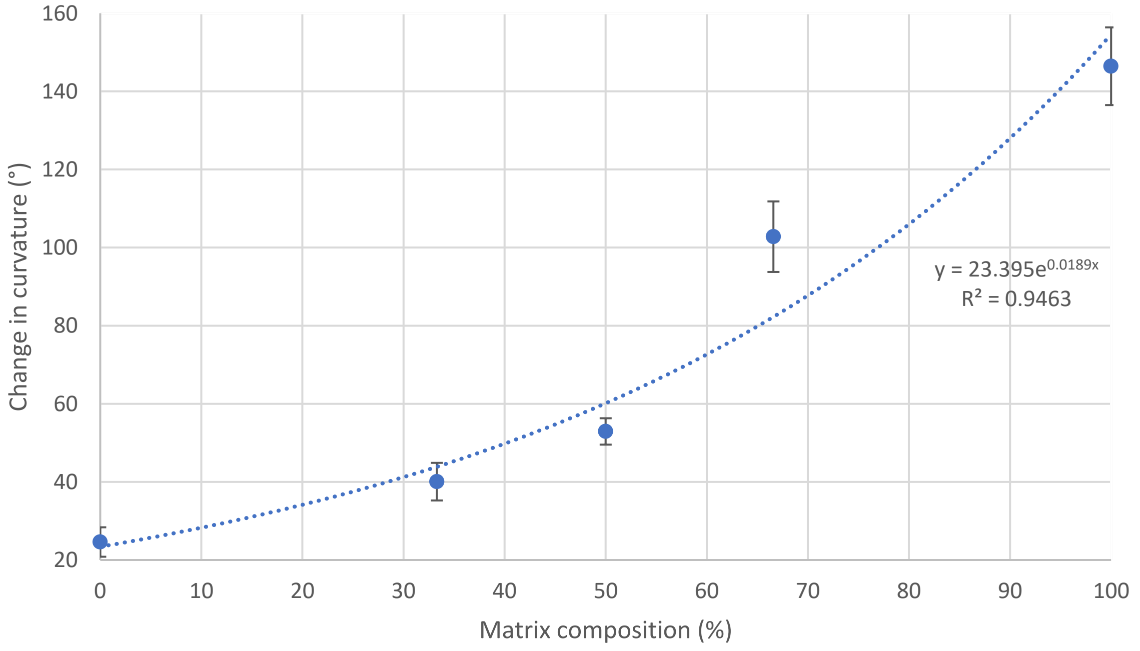

Supplementary Figure S6. Effects of Ca^{2+} concentration on matrix curvature .

Tables

Supplementary Table S1. Endothermic and exothermic events for calcium alginate, gallium alginate, calcium gallium alginate 1:1, calcium gallium alginate 1:2 and calcium gallium alginate 2:1.

**Type**	**Endothermic event**	**Exothermic event**	**Time difference between endothermic and exothermic events**	**Temperature difference °C**
Ca	30 minutes 55seconds at 175°c	33 minutes 5 seconds 186°c	3mins 50	11
Ga	30 minutes 5 seconds, 171°c	32 minutes 10 seconds, 181 °c.	2mins 10	10
1:1 caga	31 minutes 40 seconds, 179°c	32 minutes 50 seconds, 190°c	1minute 10	11
1:2 caga	29 minutes 10 seconds, 166°c	30 minutes 35 seconds, 173°c	1minute 25	7
2:1caga	31 minutes, 176°c	33 minutes 10 seconds, 187°c	2minute 10	11

Supplementary Table S2. Peak position of FTIR vibrations between calcium alginate, gallium alginate, calcium gallium alginate 1:1, calcium gallium alginate 1:2 and calcium gallium alginate 2:1.

**Vibration**	**Peak position (cm^{-1})**
O-H	3000-3650
C-H - Anomer	2850-2980
COO - Asymmetric	1500-1700
COO - Symmetric	1350-1500
CCH+OCH	1250-1350
OCO Ring	1050-1100
CO – Stretching uronic acid	920-980
